# Work Stress and Depressive Symptoms in Fishermen With a Smoking Habit: A Mediator Role of Nicotine Dependence and Possible Moderator Role of Expressive Suppression and Cognitive Reappraisal

**DOI:** 10.3389/fpsyg.2018.00386

**Published:** 2018-03-26

**Authors:** Hongjuan Jiang, Sailan Li, Juan Yang

**Affiliations:** ^1^Department of Psychology, Hainan Medical University, Haikou, China; ^2^Hainan Anning Hospital, Haikou, China

**Keywords:** work stress, depressive symptoms, nicotine dependence, expressive suppression, cognitive reappraisal, fishermen, smoking habit

## Abstract

This study examined pathways of influence between work stress, depressive symptoms, nicotine dependence, expressive suppression, and cognitive reappraisal in fishermen with smoking habits in Qionghai, Hainan province, China (N = 1068). These fishermen responded to multiple assessments a week before leaving on a deep-sea fishing trip, including a Mental Stressor Investigation Questionnaire (MSIQ), the Center for Epidemiological Studies Depression Scale (CES-D), the Russell Reason for Smoking Questionnaire (RRSQ), and an Emotion Regulation Questionnaire (ERQ). Structural equation modeling (SEM) analyses of the collected data in Mplus 7 showed that work stress and nicotine dependence were independent predictors of depressive symptoms. The relationship between work stress and depressive symptoms was found to be partially mediated by nicotine dependence and be moderated by cognitive reappraisal. The evidence suggests it advantageous to examine the need of work stress, nicotine dependence, and cognitive reappraisal when attempting to understand depressive symptoms in fishermen with a smoking habit. These findings suggest that improving nicotine dependence through work stress management and training in cognitive reappraisal could be utilized as effective modalities for improving depressive symptoms.

## Introduction

Fishing is a major traditional occupation, and fishermen often face difficult and dangerous working conditions at sea. Reported fatality rates in commercial fishing are high, relative to other occupations, in many countries, including China (214/100,000; China Fisheries Association), Australia (143/100,000; Cavalcante et al., 2017), and Poland (130.6/100,000; Jaremin et al., 1997b). Notably, Alaskan commercial fishermen in the United States have a mortality rate that is 28 times that of Alaskan workers as a whole (Thomas et al., 2001). The major causes of death at sea include vessels sinking, poor life-saving facilities, trauma, and limited access to qualified medical assistance (Jaremin et al., 1997a; Casson et al., 1998; Thomas et al., 2001; Garrone Neto et al., 2005). Fishermen are also at an elevated risk for several diseases, including musculoskeletal problems, noise-induced hearing loss, circulatory system diseases, and skin injuries (Jaremin et al., 1997a; Kaerlev et al., 2007; Percin et al., 2012). In addition, fishermen’s health can be harmed by the dysregulated serum cortisol, cumulative sleep deprivation, and self-destructive behaviors as a result of persistent overwork at sea (Allegri et al., 1996; Szymanska et al., 2006; Gander et al., 2008). As to the depression in fishermen, two small-sample surveys of fishermen with otoneurological symptoms yielded high rates of depression (23.0 and 16.7%, respectively) (Zeigelboim et al., 2014, 2015).

Work-related stressors including heavy workloads, intense time pressures, latitude in decision-making, occupational risks, and lack of support from co-workers may have negative physical and psychological health effects (Melchior et al., 2007; Meszaros et al., 2013; Fan et al., 2015). Numerous studies have examined the effects of work stress in the development of depressive symptoms. For example, Wieclaw et al. (2006) observed that employees that were exposed to work-related threats and violence were more inclined to depression. Similarly, Magnavita and Fileni (2014) study in radiologists found that awareness of work-related stress was accompanied by a marked increase in depression risk. In addition, in nursing, work stress has been shown to contribute to low self-esteem, high perceived stress, and serious occupation burnout; factors that have been related to depressive symptom levels (Lee et al., 2013; Lin et al., 2016). Although fishermen work in an unpredictable and high-risk environment, few studies have addressed the work stress and the incidence of depression among fishermen.

High rates of cigarette consumption among fisherman could be related to their high occupational stress and long work hours (Fort et al., 2010). Physically, smoking is the main cause of several serious diseases, such as emphysema, chronic bronchitis, heart disease, and lung cancer. Psychologically, the relationships between smoking, stress, and depression are complicated. On one hand, nicotine dependence has been reported to be related to occupational stress, especially in high-stress fields (John et al., 2006; Chopra et al., 2015; Sandhu et al., 2016), and the use of tobacco has been found to be employed as a coping mechanism to maintain good performance despite stress and fatigue (Ndiaye et al., 2001; Lapeyre-Mestre et al., 2004; Dawson et al., 2012). On the other hand, in a study of 197 currently smoking and employed participants, Schmidt et al. (2010) found an inverse correlation between work pressure and nicotine dependence. Meanwhile, work-associated stress was found to be unrelated to nicotine dependence among law enforcement personnel (Priyanka et al., 2016). However, the relationship between smoking and stress in fishermen is rarely reported.

Persons with more severe nicotine dependence have been found to have higher rates of major depression (Breslau et al., 1991, 1994; Son et al., 1997; Khaled et al., 2009; Pedersen and von Soest, 2009). Additionally, a 2-year study of psychiatric patients showed that nicotine dependence also affected the severity of depressive symptom (Jamal et al., 2012). Likewise, persons with severely depressive symptoms may have an increased risk of nicotine dependence (Breslau et al., 1993; McKenzie et al., 2010; Trosclair and Dube, 2010; Scherphof et al., 2013). Some studies argue that nicotine dependence can predict depressive symptoms (Brown et al., 2000; Loprinzi et al., 2014), while others insist that depressive symptoms increase a risk of nicotine dependence (Lerman et al., 1996; Currie et al., 2001; Ong and Walsh, 2001; Dierker et al., 2015; Wang et al., 2016). The recent notion that comorbidity between nicotine dependence and depressive symptoms may reflect common factors related to both outcomes is widely accepted. Two twin studies suggested that nicotine dependence-depression comorbidity was influenced by common genetic risk factors (Fu et al., 2007; Lyons et al., 2008). Edwards et al. ’ 2011 study indicated that nicotine dependence and depression shared genetic and unique environmental influences, and the shared genetic liability resulted in co-variation between nicotine dependence and depression, with the former predicting the latter (Edwards and Kendler, 2012).

Beck’s Developmental Model of Depression is one of the better models to explain the development mechanism of depression. According to Beck (2008), cognitive vulnerabilities such as dysfunctional attitudes constitute a predisposition to depression. On the basis of Beck’s theoretical model, more cognitive vulnerabilities, including emotion regulation strategies have been reported.

Depression is a disorder of impaired emotion regulation, which is to say that the emotion regulation strategies are working as crucial components in the onset and maintenance of depressive symptoms (Campbell-Sills et al., 2006a,b; Kashdan et al., 2006). A dysfunction in the neural circuitry supporting adaptive regulation, including regions of the prefrontal cortex and amygdala, may play a decisive role in vulnerability to depression (Davidson et al., 2002; Drevets, 2003). In Gross’ (1998) and Gross and Thompson (2006) process model of emotion regulation, two regulation strategies are represented as follows: Expressive suppression, referring to the inhibition of external cues to one’s internal emotional state, is associated with reduced positive affect and life satisfaction, impaired interpersonal communication, and greater negative emotion in response to negative affective stimuli (Gross and Muñoz, 1995; Butler et al., 2003; Kashdan et al., 2006), which are common risk factors for depression (Sperberg and Stabb, 1998). Cognitive reappraisal, which is involved in reframing emotion-eliciting experiences or stimuli that dampen their impact, is effective in reducing negative feelings and corresponding physiological responses in the amygdala (Ochsner et al., 2002; Gross and John, 2003; Phillips et al., 2008). Reappraisal is associated with less negative affect, increased life satisfaction (Gross and John, 2003; Garnefski et al., 2004; Garnefski and Kraaij, 2006; Kashdan et al., 2006), and less physiological arousal (Dandoy and Goldstein, 1990), all of which are protective factors for depressive symptoms.

Many studies have discussed the association between expressive suppression and depressive symptoms. On the whole, lower levels of expressive suppression appear to be protective against depression in Europeans and Chinese adolescents (Moore et al., 2008; Soto et al., 2011; Boyes et al., 2016; Sai et al., 2016). Larsen et al. (2012) study found that the depressive symptoms predicted expressive suppression in adolescents, whereas expressive suppression predicted depressive symptoms in adolescents (Zhao and Zhao, 2015; Juang et al., 2016). In addition, research also shows that emotion suppression does not always have a negative effect on depression. For example, inhibiting emotional response is effective in reducing depressive symptoms in Chinese adults (Yuan et al., 2014). Eftekhari et al. (2009) using cluster analysis found that individuals who reported low emotion regulatory style and moderate levels of suppression had the most severe depression. Moreover, expressive suppression was found to moderate the relationship between positive feelings and emotional exhaustion (Bassal et al., 2016; Norberg et al., 2016).

Cognitive reappraisal is particularly useful in stressful environments, and its use has been associated with lower rates of depression (Gross, 1998; Troy et al., 2010). In general, more use of cognitive reappraisal could lower the levels of depressive symptoms (Joormann and Gotlib, 2010). While findings indicate that cognitive reappraisal predicts depressive symptoms (Zhao and Zhao, 2015; Juang et al., 2016; Sai et al., 2016), depression has also been demonstrated to have a direct effect on cognitive reappraisal (Richmond et al., 2017). In addition, cognitive reappraisal has proposed an effective moderator between negative living condition and unfavorable outcomes. For example, Flouri and Mavroveli (2013) found that cognitive reappraisal moderated the relationship between heavy life stress and serious problem behaviors in a functionally positive manner. Similarly, Boyes et al. (2016) found that cognitive reappraisal was a moderator between adverse life experiences and psychological distress.

### The Present Study

The aim of this study is to examine how expressive suppression and cognitive reappraisal strategies interact with work stress, depressive symptoms, and nicotine dependence in a population of deep-sea fishermen. Although the deleterious effects of work stress on depressive symptoms have been observed in various other groups, including managers, military personnel, and medical professionals (Pflanz and Ogle, 2006; Magnavita and Fileni, 2014), there is rare study regarding the effects of work stress on depressive symptoms in fishermen (Thomas et al., 2001; Garrone Neto et al., 2005). Thus, information gained in this kind of research would be useful in the development of professional and pertinence intervention programs for depressive fishermen.

Both cross-sectional and longitudinal studies have suggested that work stress positively predicts nicotine dependence (Chopra et al., 2015; Sandhu et al., 2016), and that nicotine dependence and depressive symptoms share genetic and environmental risk factors (Boden et al., 2010; Edwards et al., 2011; Dierker et al., 2015). However, a mediating role of nicotine dependence upon the relationship between work stress and depressive symptoms is lacking. This is the first study to examine a potentially effect of nicotine dependence between work pressure and depressive symptoms.

Finally, expressive suppression and cognitive reappraisal have been related to depressive symptoms (Aker et al., 2014; Sai et al., 2016). Cognitive reappraisal has been reported to act as a moderator between life stress and problem behaviors in adolescents, as well as between adverse life experiences and psychological distress in high school students (Flouri and Mavroveli, 2013; Boyes et al., 2016). Meanwhile, although expressive suppression has been shown to affect stress-related symptomology, its role in the relationship between work pressure and depressive symptoms, particularly in smoking fishermen, has not been clarified.

#### Hypothesis 1: Work Stress Is Positively Related to Depressive Symptoms

Given the previous research indicating that work stress as a strong predictor of depressive symptoms (Lee et al., 2013; Magnavita and Fileni, 2014; Lin et al., 2016), we hypothesize that work stress may be a direct predictor of depressive symptoms in fishermen.

#### Hypothesis 2: Nicotine Dependence Is a Mediator Between Work Stress and Depressive Symptoms

A relationship between work stress on nicotine dependence has been extensively documented (Chopra et al., 2015; Sandhu et al., 2016). Meanwhile, associations between nicotine dependence and depressive symptoms are complicated with the former being a predictor of the latter (Edwards et al., 2011; Edwards and Kendler, 2012; Dierker et al., 2015). We therefore hypothesize that work stress may increase the risk of nicotine dependence, and subsequently increase depressive symptoms in fishermen.

#### Hypothesis 3: Expression Suppression Moderates the Relationship Between Work Stress and Depressive Symptoms

A previous study in Chinese adolescents suggests that the more use of expressive suppression means the higher levels of depressive symptoms (Sai et al., 2016). In contrast, study in Chinese adults showed that the more use of expressive suppression means the lower levels of depressive symptoms (Yuan et al., 2014). Study in students from Hong Kong indicates that the expressive suppression was not associated with depressed mood (Soto et al., 2011). Furthermore, expressive suppression is strongly related to stress-related symptoms (Moore et al., 2008; Richmond et al., 2017) and it moderates the relationship between positive feelings and emotional exhaustion (Bassal et al., 2016). Then, we proposed that expressive suppression may not have direct relationship with depressive symptoms, but it may be a moderator between work stress and depressive symptoms in fishermen.

#### Hypothesis 4: Cognitive Reappraisal Moderates the Relationship Between Work Stress and Depressive Symptoms

Cognitive reappraisal can affect stress-related symptoms and has a close relationship with depressive symptoms (e.g., Juang et al., 2016; Richmond et al., 2017). Cognitive reappraisal can act as a moderator between negative living conditions and psychological/behavioral problems (Flouri and Mavroveli, 2013; Boyes et al., 2016). Thus, we hypothesized that cognitive reappraisal may act as a moderator between work stress and depressive symptoms in fishermen.

## Materials and Methods

### Participants

This study was approved by the Ethics Committee of Hainan Medical University. Commercial fishermen were recruited from Mandarin Chinese in Tanmen Town, Qionghai, China. Signed consent forms were obtained from over 95% fishermen who expressed interest in participating in the study. Interviews were conducted about a week before the participants went out for deep sea fishing. A total of 1,068 fishermen with a smoking habit which provided valid data across all study variables were employed in the current analysis. All the participants completed the questionnaires in the normal state, without hunger, fatigue, diseases and other things like that. Detailed demographic information shows in **Table 1**.

**Table 1:** Demographic characteristics of the present study cohort of Fishermen.

Variable	Frequency
Mean age ± SD (range), years	38.05 ± 10.75 (18–67)
Level of education completed	
Elementary school or less	29.8%
Middle school	63.4%
Technical secondary school	1.9%
High school or higher	5.0%
Time employed in fishing	
<1 year	8.3%
1–3 years	11.9%
3–5 years	8.9%
>5 years	70.9%
Marital status	
Never married	27.6%
Married	70.4%
Divorced	2.0%
Religion	
None	76.9%
Christianity	2.2%
Buddhism	15.8%
Taoism	4.0%
Other	1.0%

### Procedures

A local project coordinator collected the signed consent forms. The consenting fishermen completed each of the following four questionnaires: Mental Stressor Investigation Questionnaire (MSIQ); Center for Epidemiological Studies Depression Scale (CES-D)-Chinese version; Russell Reason for Smoking Questionnaire (RRSQ); and Emotion Regulation Questionnaire (ERQ).

### Measures

#### Work Stress

Stress exposure was measured with the MSIQ (Yu et al., 2014). This scale was developed to assess work stress among naval ship crewmembers. It has a strong reliability score of 0.97 and validity of 0.75–0.96. In the present study, a short form of the scale was used, which was comprised of 36 items that addressed two factors: ship environment, and relations between work and interpersonal. The score for each item ranged from 1 (not at all) to 5 (almost all the time), with higher scores represent higher stress levels. The Cronbach’s α of the total scale was 0.95.

#### Depressive Symptoms

Depressive symptoms were investigated with the Chinese version of the highly reliable and widely used CES-D (Radloff, 1977; Yang et al., 2004). Each item of the CES-D scale was scored 0 (rarely) to 3 (all of the time). The Chinese version of CES-D contained 20 items that reflected four observable variables: depressive, somatic, positive, and interpersonal variable (Makambi et al., 2009). The full-scale scores were scored 0–60. A score of 16 is the standard cut-off score for depression. The internal consistency of this scale was 0.91.

#### Nicotine Dependence

Dependence on smoking was assessed with the Russell’s Smoking Motivation Questionnaire (RRSQ) (Russell et al., 1974), which was derived from the Chinese version of RRSQ (Wang et al., 1999). The questionnaire contains eight subscales and 24 items with each item being scored 0 (not at all) to 3 (very much so). Five (Psychological image, Hand-mouth, Indulgent, Sedative, and Stimulation) of the eight subscales are used independently. The Addictive, Automatic, and Auxiliary subscale can be used independently or together, such as for nicotine dependence. A score <6 was classified as no dependence, a score between 6 and 20 was classified as dependence, and a score >20 indicated heavy dependence. In the current investigation, the reduction subscale demonstrated a good internal consistency (α = 0.92).

#### Expressive Suppression and Cognitive Reappraisal

The Chinese version of the ERQ consisted of 10 items that reflect two factors: expressive suppression (4 items) and cognitive reappraisal (6 items) (Wang et al., 2007). Each item of the ERQ was scored 1 (completely disagree) to 7 (completely agree). The Chinese version of the ERQ showed good validation in Chinese individuals (Wang et al., 2007) with a Cronbach’s α of 0.84 and 0.90 for expressive suppression and cognitive reappraisal, respectively.

### Statistical Analyses

Mean values were reported with standard deviations (SDs). Data were analyzed using SPSS 21 (IBM Corp., Armonk, NY, United States). The alpha value was set at 0.05.

Associations among work stress, nicotine dependence, expressive suppression, cognitive reappraisal, and depressive symptoms were analyzed with structural equation modeling (SEM) in Mplus 7 (Muthén and Muthén, 1998–2010, Los Angeles, CA, United States).

The TECH13 option was used in conjunction with TYPE = MIXTURE to request two sided tests of model fit for multivariate skewness and kurtosis (Mardia’s measure of multivariate kurtosis).

The comparative fit index (CFI), standardized root mean square residual (SRMR), and root mean square error of approximation (RMSEA) were used to determine goodness of fit with a cut-off value of >0.95, <0.09, and <0.08, respectively (Iacobucci, 2010). Additionally, Akaike information criterion (AIC) and Bayesian information criterion (BIC) values were calculated as indices of relative quality.

The BOOTSTRAP option was used in conjunction with both the MODEL CONSTRAINT option and the CINTERVAL (BCBOOTSTRAP) option to obtain indirect effects bootstrapped standard errors and bootstrap confidence intervals.

Latent moderated structural (LMS) equations were used in the latent moderation model.

The LOOP option was used together with the PLOT option to make plots. The variable total direct effect was on the *y*-axis and the moderating variable (i.e., expressive suppression or cognitive reappraisal) was on the *x*-axis. The lower, upper, and incremental values of the moderating variable were 4/6, 28/42, and 2/3, respectively.

## Results

### Descriptive Analyses

Descriptive variables were assessed directly with psychometric instruments (**Table 2**). Most of the fishermen had a normal mood (mean total CES-D score, 5.51 ± 7.11; median score, 3). Specifically, a total of 91.9% of the fishermen in this study scored under 16, 7.2% of the fishermen scored between 16 and 32, and 0.9% scored higher than 32. None of the demographic factors examined were related to CES-D score, work stress, nicotine dependence, expressive suppression, or cognitive reappraisal levels, indicating that SEM could be conducted without considering the demographic factors.

**Table 2:** Descriptive statistics for all observable variables.

*Instrument* observable variable	Mean	*SD*
*MSIQ*		
Ship environment	43.85	17.40
Work and interpersonal relations	13.79	4.94
RRSQ		
Addictive	1.45	2.05
Automatic	1.22	1.51
Auxiliary	2.14	2.10
*CES-D*		
Depressed	1.82	2.32
Somatic	2.22	2.77
Positive	1.09	1.67
Interpersonal	0.37	0.66
*ERQ*		
Expressive suppression	15.02	6.55
Cognitive reappraisal	24.04	9.97

*MSIQ, Mental Stressor Investigation Questionnaire; RRSQ, Russell Reason for Smoking Questionnaire; CES-D, Center for Epidemiological Studies Depression Scale; ERQ, Emotion Regulation Questionnaire.*

The percentage of data missing for expressive suppression, cognitive reappraisal, and depressive symptoms was 0.4, 0.3, and 0.1%, respectively. For each variable with missing data, the data group and the absent group had no significant difference in the other indicators (*t* = 0.3–1, *p* > 0.05). The results of a multivariate *t*-test suggested that all missing data were missed *at random* and that the full-information maximum likelihood approach was suitable for managing the missing data.

The multivariate non-normality test showed that testing for both multivariate skewness (sample value = 1293.093, mean = 4.622, standard deviation = 0.228, *p* < 0.001) and kurtosis (sample value = 1833.143, mean = 287.715, standard deviation = 1.520, *p* < 0.001) were statistically significant, indicating violation of multivariate normality assumption. The rescaling-based maximum likelihood robust (MLR) estimator, would be proposed to deal with non-normal data.

### Main Analyses

The zero-order correlations (*r*-values) among the latent (inferred) variables were presented in **Table 3**. Notably, both work stress (inferred from MSIQ scores) and nicotine dependence (inferred from RRSQ scores) had highly significant associations with depressive symptoms (inferred from CES-D scores), while expressive suppression and cognitive reappraisal (both inferred from ERQ sub-scores) were related to each other. In addition, work stress, nicotine dependence, expressive suppression, and cognitive reappraisal were all positively related to each other.

**Table 3:** Zero-order correlations among latent variables.

Latent variable (inferred from)	1	2	3	4	5
(1) Work stress (MSIQ)	1	0.257^∗∗∗^	0.124^∗∗∗^	0.137^∗∗∗^	0.580^∗∗∗^
(2) Nicotine dependence (RRSQ)	–	1	0.166^∗∗∗^	0.182^∗∗∗^	0.316^∗∗∗^
(3) Expressive suppression (ERQ)	–	–	1	0.770^∗∗∗^	0.056
(4) Cognitive reappraisal (ERQ)	–	–	–	1	0.027
(5) Depressive symptoms (CES-D)	–	–	–	–	1

*^∗∗∗^p < 0.001; MSIQ, Mental Stressor Investigation Questionnaire; RRSQ, Russell Reason for Smoking Questionnaire; CES-D, Center for Epidemiological Studies Depression Scale; ERQ, Emotion Regulation Questionnaire.*

The SEM consisted of two parts: a measurement model and a structural model. We first tested the relationships between observable and latent variables in a measurement model. The model fit information of each latent variable in the measurement model was presented in **Table 4**. All indicators were accepted.

**Table 4:** Model fitting information for the measurement model.

Latent variable (inferred from)	X^2^	*df*	TLI	CFI	AIC	BIC	SRMR	RMSEA (90% CI)
Work stress (MSIQ)	0	0	1	1	19587.885	19632.647	0	0
Nicotine dependence (RRSQ)	0	0	1	1	12495.807	12540.535	0	0
Depressive symptoms (CES-D)	4.480	2	0.999	0.997	14648.174	14707.811	0.007	0.034 (0.000, 0.078)
Expressive suppression (ERQ)	3.606	1	0.990	0.998	16456.824	16521.443	0.006	0.049 (0.000, 0.109)
Cognitive reappraisal (ERQ)	0	0	1	1	15476.987	15521.723	0	0

*CFI, comparative fit index; AIC, Akaike information criterion; BIC, Bayesian information criterion; SRMR, standardized root mean square residual; RMSEA, root mean square error of approximation; MSIQ, Mental Stressor Investigation Questionnaire; RRSQ, Russell Reason for Smoking Questionnaire; CES-D, Center for Epidemiological Studies Depression Scale; ERQ, Emotion Regulation Questionnaire.*

**FIGURE 1:**
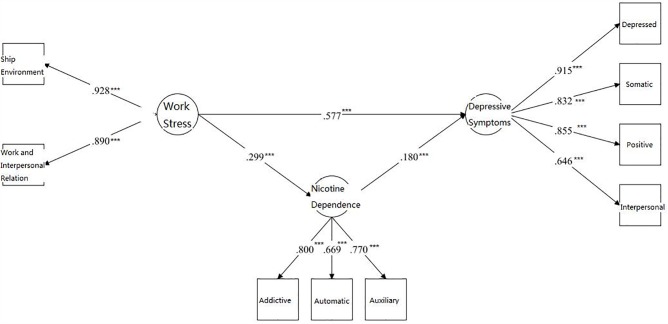
Path analysis describing the associations between work stress, depressive symptoms, and nicotine dependence among fishermen with a smoking habit. ^∗∗∗^*p* < 0.001.

Next, we employed the structural model component of SEM to test whether work stress can predict depressive symptoms (section “*Hypothesis 1*”) and whether nicotine dependence level can act as a mediator in the relationship between work stress and depressive symptoms (section “*Hypothesis 2*”). All indices showed excellent model fitness (CFI = 0.981, RMSEA = 0.048, SRMR = 0.033, AIC = 40919.940, and BIC = 41069.034). All factor loadings for work stress, nicotine dependence, and depressive symptoms were significant (*p* < 0.001), suggesting that the measurement model was acceptable. A visual depiction of the model was presented in **Figure 1**. A significant indirect path from work stress to level of depressive symptoms was observed via nicotine dependence (β = 0.054, 95% CI 0.032–0.089; *p* < 0.001), which accounted for 8.56% of the total effect.

Based on our findings from the above, we tested whether the expressive suppression served as a moderator between work stress and depressive symptoms (section “*Hypothesis 3*”) by using LMS. The original model estimation did not terminate normally due to a change in the log likelihood during the last step with LMS equations. Therefore, we employed the product indicator approach and obtained model fit indices indicating that the model estimation terminated normally and could not be accepted (only providing SRMR = 0.097, AIC = 57406.964, BIC = 57764.789). The regression coefficient from expressive suppression to depressive symptoms was -0.116 (*p* = 0.110). Work stress × expressive suppression interaction was not predictive for depressive symptoms (β = -0.117, *p* = 0.069).

**FIGURE 2:**
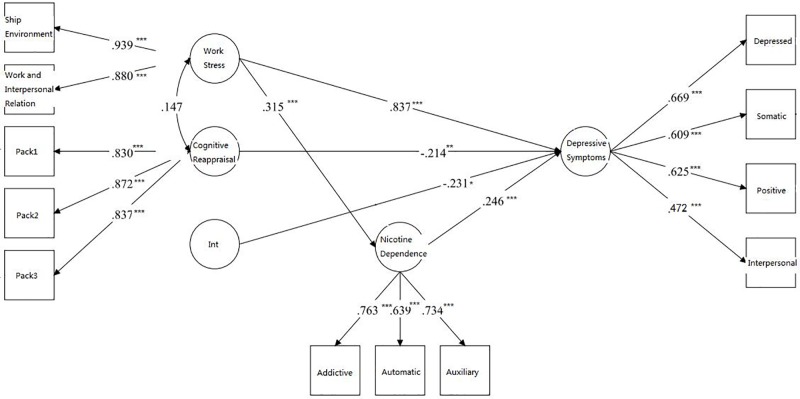
Path analysis describing the associations between work stress, depressive symptoms, nicotine dependence, and cognitive reappraisal among fishermen with a smoking habit. The item parceling (dividing by item content) and cognitive reappraisal variable had three packs. Int = Work Stress ^∗^ Cognitive Reappraisal. Parameters are standardized. ^∗∗^*p* < 0.01, ^∗∗∗^*p* < 0.001.

**FIGURE 3:**
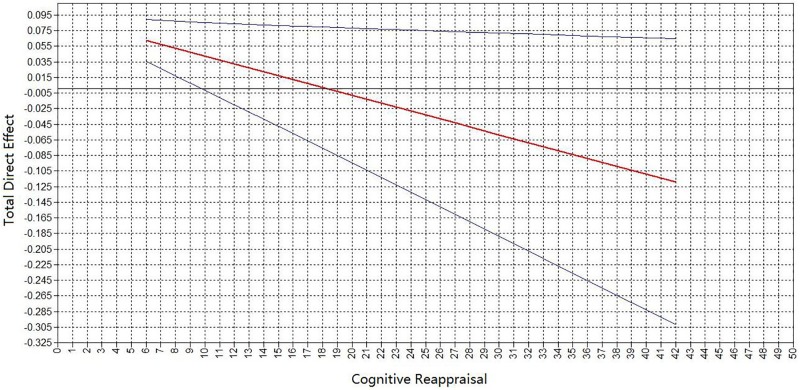
Graphical representation of cognitive reappraisal moderation of a total direct effect between work stress and depressive symptoms. Predicted slopes for the relationship between work stress and depressive symptoms in relation to cognitive reappraisal (score range, 6–42) for Chinese fishermen with a smoking habit. The middle red line represents the total direct effect between work stress and depressive symptoms; such that the steepness of the slope reflects the strength of the moderating influence (a slope of 0 would indicate no moderating effect). Note that the higher the emotion regulation score for cognitive reappraisal (*x* axis value), the weaker the total direct effect is (*y* axis value). The area between the upper and lower (blue) lines represents the CI of the total direct effect.

Finally, SEM was conducted to re-test the hypotheses that work stress can predict depressive symptoms (section “*Hypothesis 1*”) and that nicotine dependence serves as a mediator in the relationship between the work stress and depressive symptom (section “*Hypothesis 2*”). Whether cognitive reappraisal may serve as a moderator (section “*Hypothesis 4*”) was investigated using LMS. The model fit indices were as follows: AIC = 29267.766 and BIC = 29476.497. All factor loadings for the latent variable indicators were significant (*p* < 0.001) (**Figure 2**). The moderating effect was -0.231 (*p* = 0.025). Work stress, the level of nicotine dependence, and cognitive reappraisal significantly predicted depressive symptoms (β = 0.837, β = 0.246, and β = -0.214, respectively; all *p* < 0.01). There was also a significantly direct association between work stress and level of nicotine dependence (β = 0.315, *p* < 0.001). A significantly indirect path from work stress to the level of depressive symptoms was observed via nicotine dependence (β = 0.077, *p* < 0.001), accounting for 11.27% of the total effect. The total effect of work pressure on depression was reduced from β = 0.683 to a direct effect of β = 0.606. The work stress × cognitive reappraisal interaction was a significant predictor of depressive symptoms (β = -0.231, *p* = 0.025), indicating that cognitive reappraisal moderated the direct association between work stress and depressive symptoms. The moderating influence of cognitive reappraisal on the direct association between work stress and depressive symptoms is summarized in **Figure 3**. Note that the strength of the work stress-depressive symptoms direct effect was lessened with increasing levels of cognitive reappraisal.

## Discussion

In the present study, we examined the relationships between work stress, nicotine dependence, expressive suppression, cognitive reappraisal, and depressive symptoms in 1068 Chinese fishermen with smoking habits. Path analysis modeling indicated that work stress affects depressive symptoms directly and also affects depressive symptoms indirectly via nicotine dependence. The connection between work stress and depressive symptoms was moderated by cognitive reappraisal. Our findings suggest that interventions to reduce work stress have the potential to improve mental health in fishermen. In addition, improvements in work stress that may help reduce nicotine dependence may also, in turn, decrease depressive mood. Moreover, the adaptive cognitive reappraisal strategies may help fishermen endure the stresses associated with their job.

### Depressive Symptoms in Smoking Fishermen

It has been proposed that psychological problems often occur in fishermen, who work in an environment that presents risks associated with nature and boat travel, as well as stresses related to peer relationships (Jaremin et al., 1997b; Casson et al., 1998; Thomas et al., 2001; Garrone Neto et al., 2005). Hence, we hypothesized that our study population (smoking fishermen) may have serious depressive symptoms. Unexpectedly, in the present study, the prevalence of depressive symptoms (8.1%) in fishermen with a smoking habit is lower than that in previous reports (Zeigelboim et al., 2014, 2015). More surprisingly, it is even lower than that in the general population, as the literature suggests that approximately 18% of middle-aged Chinese men in Hong Kong are affected by depressive symptoms (Wong et al., 2006); the reported prevalence of depression in male freshmen is 24.8% in Peking and 36.1% in Hong Kong (Song et al., 2008). It seems that the fishermen with a smoking habit in Hainan Province in China were not that serious in depressive symptoms. It is possible that the policies encouraging and supporting the development of fishing in China benefit the living and working conditions of fishermen, including subsides for diesel fuel, the renewal and remolding of fishing boat, and so on. Also, the longevity and well-being of the Hainan province population were counteracting factors that protected the fisherman from depression. Prospective studies are required to assess whether this finding is an accidental phenomenon.

### Work Stress and Depressive Symptoms

In this study, work stress in fishermen is related to their work environments and interpersonal relationships, which have been related to the development of depressive symptoms. Our findings were in line with our hypothesis that high scores on work stress may lead to increased levels of depressive symptoms and consistent with prior studies (Lee et al., 2013; Magnavita and Fileni, 2014; Lin et al., 2016). As deep sea fishermen are particularly susceptible to risk from the prolonged hours of continuous work and constant vigilance, such as increased risk of falling, machinery entanglements, and being hit by objects (Gander et al., 2008), it suggests that the improvement in living and working conditions might be the first choice to ensure safety in sea. Moreover, as fishermen often face uncertainty regarding unpredictable aquatic animals and the possibility of their vessel capsizing or sinking (Garrone Neto et al., 2005), it is necessary to enhance their interpersonal skills so as to improve their cooperation capability. Further research should focus on potential moderators/mediators between work stress and depressive symptoms.

### Work Stress, Nicotine Dependence, and Depressive Symptoms

Unlike the results of Schmidt et al. (2010) and Priyanka et al. (2016), who found that work stress had no connection or negative connection with nicotine dependence, this study was consistent with most of the research showing that work stress positively impacted nicotine dependence (John et al., 2006; Chopra et al., 2015; Sandhu et al., 2016). With respect to the relationship between nicotine dependence and depressive symptoms, the former directly predicted the later, which is consistent with prior studies (Edwards et al., 2011; Edwards and Kendler, 2012; Dierker et al., 2015). As a whole, the present findings demonstrated that nicotine dependence plays a role in linking work stress to depressive symptoms. Commonly, people who are stressed in work are prone to serious nicotine dependence (Chopra et al., 2015), and this phenomenon is especially prominent in high-risk occupations (John et al., 2006). Many fishermen, like workers in other occupations, regard smoking as an effective mean to relieve work stress, especially when their work extends into the night (Priyanka et al., 2016). Smokers with mild to moderate nicotine dependence have also been reported to exhibit an increased risk of depressive symptoms (Manley et al., 2009; Boden et al., 2010; Ashor, 2013). In the present study, nicotine dependence was found to partially mediate the association between work stress and depressive symptoms. However, this indirect effect was weaker than the direct interaction between work stress and depressive symptoms. These findings suggested that the effective work stress management could not only decrease depressive symptoms directly but could also indirectly relieve it through nicotine dependence. In future, longitudinal design should be employed to examine whether depressive symptoms could predict nicotine dependence (Lerman et al., 1996; Currie et al., 2001; Ong and Walsh, 2001; Dierker et al., 2015; Wang et al., 2016), and even more, whether nicotine dependence link the relationship between depressive symptoms and work stress.

### Work Stress, Expressive Suppression, and Depressive Symptoms

Expressive suppression, wherein behavioral expression regarding an emotional experience is inhibited, can contribute to or exacerbate stress-related symptoms (Gross, 1998; Moore et al., 2008; Richmond et al., 2017). However, in this study, expressive suppression not only did not predict depressive symptoms but also did not moderate the relationship between work stress and depressive symptoms. This suggests that the translation of work stress to depressive symptoms appears to be equally robust for those with and without expressive suppression. Firstly, it is possible that the effects of expressive suppression may not be as strong as once thought. For example, Masumoto et al. (2016) study of Japanese participants (age range, 20–70 years old) found that expressive suppression has a smaller impact on mood than cognitive reappraisal. Similarly, Barrault et al. (2017) study of regular online poker players found that expressive suppression was not linked with depression. Secondly, these negative findings could be related to culture; the correlation between expressive suppression and health problems in Asian subjects is not as strong as in Western subjects (Hu et al., 2014). Thirdly, previous studies in Chinese adolescents showed that expressive suppression had a positive relationship with depressive symptoms (Zhao and Zhao, 2015; Sai et al., 2016), but study in Chinese adults shows no relationship between them (Yuan et al., 2014). Although the present study did not confirm our hypothesis, it helps to expand our knowledge about expressive suppression, especially its relationship with work stress and depressive symptoms. We also provide new information about the different roles of expressive suppression and cognitive reappraisal in the same context. In future, if possible, more research needs to discover the relationship between expressive suppression and depressive symptoms in Chinese adults, with the focus on exploring the indirect effect or intermediate variable between them.

### Work Stress, Cognitive Reappraisal, and Depressive Symptoms

Finally, we observed that the relation between work stress and depressive symptoms was weakened in those subjects who reported higher (at least average) levels of cognitive reappraisal. This means that the fishermen with a smoking habit tended not engage in cognitive reappraisal and were more inclined to depressive symptoms than those who did cognitive reappraisal. This was consistent with prior studies, persons who struggle with regulating their emotions have been reported to have more negative responses to stressors, and the adoption of effective emotion-adjusting strategies has been shown to be related to lesser reporting of depressive symptoms (Betts et al., 2009; Ford et al., 2014). The present findings suggest the possibility that cognitive reappraisal training might have a direct positive impact on the relationship between work stress and depressive symptoms in fishermen. Prior research have proved the efficiency of intervention on cognitive reappraisal, for example, the cognitive behavioral therapy (enhancing cognitive reappraisal) could reduce the negative emotion problem by modifying cognitive reappraisal-related prefrontal cortex neural signal magnitude (Goldin et al., 2013); the body-mind relaxation meditation induction could help depression patients construct reappraisal strategies (Chen et al., 2015); and the brief mindful emotion awareness and cognitive reappraisal interventions could lead to large reductions in self-reported levels of negative emotion problems (Bentley et al., 2017). Thus, improvements in cognitive reappraisal can be an effective way to mitigate the impact of work stress on depressive symptoms.

### Limitations

There were several limitations associated with the current study. Firstly, this study used cross-sectional data in relation validation analyses. The causal attributions or determinations of the directionality of relationships between the variables could not be established. Studies with a longitudinal or intervention-based design are needed to reveal causes of depressive symptoms. Second, the fishermen that participated in this study all lived in the same locality. Therefore, the sample may not represent other populations accurately. Finally, our response rate was 97%. The omission of the remaining 3% may cause biases. For example, participants who completed the survey might have better interpersonal skills, better outlooks, and more positive emotional experiences than those who did not participate in the study. Notwithstanding, the sample size in this study is sufficiently large enough to extend our understanding of the relationships between work stress, nicotine dependence, expressive suppression, cognitive reappraisal, and depressive symptoms in a population of fishermen who smoke. By studying a large sample of fishermen with highly valid self-rating questionnaires, including an assessment developed for crews of naval ships, the results obtained provide valuable insights into a high-risk, high mortality rate occupation.

## Author Contributions

SL collected the data and wrote the manuscript. HJ analyzed the data and wrote the manuscript. JY revised the writing, supervised the study, and got fund for the project.

## Conflict of Interest Statement

The authors declare that the research was conducted in the absence of any commercial or financial relationships that could be construed as a potential conflict of interest.

## References

[B1] Aker M. Harmer C. Landro N. I. (2014). More rumination and less effective emotion regulation in previously depressed women with preserved executive functions. *BMC Psychiatry* 14:334. 10.1186/s12888-014-0334-4 25427967 PMC4253635

[B2] Allegri F. Passarello B. Orrù G. Coppola A. Antona A. Cannizzaro E. (1996). Effects of prolonged work on "deep-sea" fishermen: influence of blood cortisol, prolactinemia and urinary catecholamines. *G. Ital. Med. Lav.* 18 101–105. 9312439

[B3] Ashor A. W. (2013). Inverted U shaped effect of nicotine on the severity of depressive symptoms: a population-based survey. *J. Young Pharm.* 5 60–63. 10.1016/j.jyp.2013.06.004 24023456 PMC3758084

[B4] Barrault S. Bonnaire C. Herrmann F. (2017). Anxiety, depression and emotion regulation among regular online poker players. *J. Gambl. Stud.* 33 1039–1050. 10.1007/s10899-017-9669-3 28105539

[B5] Bassal C. Czellar J. Kaiser S. Dan-Glauser E. S. (2016). Relationship between emotions, emotion regulation, and well-being of professional caregivers of people with dementia. *Res. Aging* 38 477–503. 10.1177/0164027515591629 26092207

[B6] Beck A. T. (2008). The evolution of the cognitive model of depression and its neurobiological correlates. *Am. J. Psychiatry* 165 969–977. 10.1176/appi.ajp.2008.08050721 18628348

[B7] Bentley K. H. Nock M. K. Sauer-Zavala S. Gorman B. S. Barlow D. H. (2017). A functional analysis of two transdiagnostic, emotion-focused interventions on nonsuicidal self-injury. *J. Consult. Clin. Psychol.* 85 632–646. 10.1037/ccp0000205 28394171 PMC5440198

[B8] Betts J. Gullone E. Allen J. S. (2009). An examination of emotion regulation, temperament, and parenting style as potential predictors of adolescent depression risk status: a correlational study. *Br. J. Dev. Psychol.* 27 (Pt 2), 473–485. 10.1348/026151008X314900 19998542

[B9] Boden J. M. Fergusson D. M. Horwood L. J. (2010). Cigarette smoking and depression: tests of causal linkages using a longitudinal birth cohort. *Br. J. Psychiatry* 196 440–446. 10.1192/bjp.bp.109.065912 20513853

[B10] Boyes M. E. Hasking P. A. Martin G. (2016). Adverse Life experience and psychological distress in adolescence: moderating and mediating effects of emotion regulation and rumination. *Stress Health* 32 402–410. 10.1002/smi.2635 25764473

[B11] Breslau N. Kilbey M. Andreski P. (1991). Nicotine dependence, major depression, and anxiety in young adults. *Arch. Gen. Psychiatry* 48 1069–1074. 10.1001/archpsyc.1991.01810360033005 1845224

[B12] Breslau N. Kilbey M. M. Andreski P. (1993). Nicotine dependence and major depression. New evidence from a prospective investigation. *Arch. Gen. Psychiatry* 50 31–35. 10.1001/archpsyc.1993.01820130033006 8422219

[B13] Breslau N. Kilbey M. M. Andreski P. (1994). DSM-III-R nicotine dependence in young adults: prevalence, correlates and associated psychiatric disorders. *Addiction* 89 743–754. 10.1111/j.1360-0443.1994.tb00960.x 8069175

[B14] Brown C. Madden P. A. Palenchar D. R. Cooper-Patrick L. (2000). The association between depressive symptoms and cigarette smoking in an urban primary care sample. *Int. J. Psychiatry Med.* 30 15–26. 10.2190/NY79-CJ0H-VBAY-5M1U 10900558

[B15] Butler E. A. Egloff B. Wilhelm F. H. Smithm N. C. Erickson E. A. Gross J. J. (2003). The social consequences of expressive suppression. *Emotion* 3 48–67. 10.1037/1528-3542.3.1.48 12899316

[B16] Campbell-Sills L. Barlow D. H. Brown T. A. Hoffman S. G. (2006a). Acceptability and suppression of negative emotion in anxiety and mood disorders. *Emotion* 6 587–595. 10.1037/1528-3542.6.4.587 17144750

[B17] Campbell-Sills L. Barlow D. H. Brown T. A. Hoffman S. G. (2006b). Effects of suppression and acceptance on emotional responses of individuals with anxiety and mood disorders. *Behav. Res. Ther.* 44 1251–1263. 10.1016/j.brat.2005.10.001 16300723

[B18] Casson F. F. Zucchero A. Boscolo Bariga A. Malusa E. Veronese C. Boscolo Rizzo P. (1998). Work and chronic health effects among fishermen in Chioggia, Italy. *G. Ital. Med. Lav. Ergon.* 20 68–74. 9658237

[B19] Cavalcante E. S. Pessoa J. M. J. Freire I. L. Cavalcante C. A. Miranda F. A. (2017). Social representations of fishermen with spinal cord injury: impacts and life trajectory. *Rev. Bras. Enferm.* 70 139–145. 10.1590/0034-7167-2016-0436 28226053

[B20] Chen F. Lv X. Fang J. Yu S. Sui J. Fan L. (2015). The effect of body-mind relaxation meditation induction on major depressive disorder: a resting-state fMRI study. *J. Affect. Disord.* 183 75–82. 10.1016/j.jad.2015.04.030 26001666

[B21] Chopra A. Lakhanpal M. Gupta N. Suri V. Kaur G. Bhudhiraja S. (2015). The influence of occupational stress factors on nicotine dependence among students of health and nonhealth care professional colleges. *Niger Med. J.* 56 349–352. 10.4103/0300-1652.170391 26778887 PMC4698851

[B22] Currie S. R. Hodgins D. C. el-Guebaly N. Campbell W. (2001). Influence of depression and gender on smoking expectancies and temptations in alcoholics in early recovery. *J. Subst. Abuse* 13 443–458. 10.1016/S0899-3289(01)00090-6 11775075

[B23] Dandoy A. C. Goldstein A. G. (1990). The use of cognitive appraisal to reduce stress reactions: a replication. *J. Soc. Behav. Pers.* 5 275–285.

[B24] Davidson R. J. Pizzagalli D. Nitschke J. B. Putnam K. (2002). Depression: perspectives from affective neuroscience. *Annu. Rev. Psychol.* 53 545–574. 10.1146/annurev.psych.53.100901.135148 11752496

[B25] Dawson A. P. Cargo M. Stewart H. Chong A. Daniel M. (2012). "I know it’s bad for me and yet I do it": exploring the factors that perpetuate smoking in Aboriginal Health Workers–a qualitative study. *BMC Health Serv. Res.* 12:102. 10.1186/1472-6963-12-102 22533609 PMC3394210

[B26] Dierker L. Rose J. Selya A. Piasecki T. M. Hedeker D. Mermelstein R. (2015). Depression and nicotine dependence from adolescence to young adulthood. *Addict. Behav.* 41 124–128. 10.1016/j.addbeh.2014.10.004 25452055 PMC4314348

[B27] Drevets W. C. (2003). Neuroimaging abnormalities in the amygdala in mood disorders. *Ann. N. Y. Acad. Sci.* 985 420–444. 10.1111/j.1749-6632.2003.tb07098.x 12724175

[B28] Edwards A. C. Kendler K. S. (2012). A twin study of depression and nicotine dependence: shared liability or causal relationship? *J. Affect. Disord.* 142 90–97. 10.1016/j.jad.2012.03.048 22901332 PMC3483438

[B29] Edwards A. C. Maes H. H. Pedersen N. L. Kendler K. S. (2011). A population-based twin study of the genetic and environmental relationship of major depression, regular tobacco use and nicotine dependence. *Psychol. Med.* 41 395–405. 10.1017/S0033291710000589 20406522 PMC3016459

[B30] Eftekhari A. Zoellner L. A. Vigil S. A. (2009). Patterns of emotion regulation and psychopathology. *Anxiety Stress Coping* 22 571–586. 10.1080/10615800802179860 19381989 PMC3234115

[B31] Fan L. B. Blumenthal J. A. Watkins L. L. Sherwood A. (2015). Work and home stress: associations with anxiety and depression symptoms. *Occup. Med.* 65 110–116. 10.1093/occmed/kqu181 25589707 PMC4402380

[B32] Flouri E. Mavroveli S. (2013). Adverse life events and emotional and behavioural problems in adolescence: the role of coping and emotion regulation. *Stress Health* 29 360–368. 10.1002/smi.2478 23281019

[B33] Ford B. Q. Mauss I. B. Troy A. S. Smolen A. Hankin B. (2014). Emotion regulation moderates the risk associated with the 5-HTT gene and stress in children. *Emotion* 14 930–939. 10.1037/a0036835 24866526 PMC4172506

[B34] Fort E. Massardier-Pilonchery A. Bergeret A. (2010). Psychoactive substances consumption in French fishermen and merchant seamen. *Int. Arch. Occup. Environ. Health* 83 497–509. 10.1007/s00420-009-0473-y 19885671

[B35] Fu Q. Heath A. C. Bucholz K. K. Lyons M. J. Tsuang M. T. True W. R. (2007). Common genetic risk of major depression and nicotine dependence: the contribution of antisocial traits in a United States veteran male twin cohort. *Twin Res. Hum. Genet.* 10 470–478. 10.1375/twin.10.3.470 17564505 PMC3254140

[B36] Gander P. van den Berg M. Signal L. (2008). Sleep and sleepiness of fishermen on rotating schedules. *Chronobiol. Int.* 25 389–398. 10.1080/07420520802106728 18533331

[B37] Garnefski N. Kraaij V. (2006). Relationships between cognitive emotion regulation strategies and depressive symptoms: a comparative study of five specific samples. *Pers. Individ. Differ.* 2006 1659–1669. 10.1016/j.paid.2005.12.009

[B38] Garnefski N. Teerds J. Kraaij V. Legerstee J. van den Kommer T. (2004). Cognitive emotion regulation strategies and depressive symptoms: differences between males and females. *Pers. Individ. Differ.* 2004 267–276. 10.1016/S0191-8869(03)00083-7

[B39] Garrone Neto D. Cordeiro R. C. Haddad V. Jr. (2005). [Work-related accidents in traditional fishermen from the Medium Araguaia River region, Tocantins, Brazil]. *Cad. Saude. Publica* 21 795–803. 10.1590/S0102-311X2005000300013 15868037

[B40] Goldin P. R. Ziv M. Jazaieri H. Hahn K. Heimberg R. Gross J. J. (2013). Impact of cognitive behavioral therapy for social anxiety disorder on the neural dynamics of cognitive reappraisal of negative self-beliefs: randomized clinical trial. *JAMA Psychiatry* 70 1048–1056. 10.1001/jamapsychiatry.2013.234 23945981 PMC4141477

[B41] Gross J. J. (1998). Antecedent- and response-focused emotion regulation: divergent consequences for experience, expression, and physiology. *J. Pers. Soc. Psychol.* 74 224–237. 10.1037/0022-3514.74.1.224 9457784

[B42] Gross J. J. John O. P. (2003). Individual differences in two emotion regulation processes: implications for affect, relationships, and well-being. *J. Pers. Soc. Psychol.* 85 348–362. 10.1037/0022-3514.85.2.348 12916575

[B43] Gross J. J. Muñoz R. F. (1995). Emotion regulation and mental health. *Clin. Psychol. Sci. Pract.* 2 151–164. 10.1111/j.1468-2850.1995.tb00036.x

[B44] Gross J. J. Thompson R. A. (2006). “Emotion regulation: conceptual foundations,” in *Handbook of Emotion Regulation* ed. Gross J. J. (New York, NY: The Guilford Press) 3–26.

[B45] Hu T. Zhang D. Wang J. Mistry R. Ran G. Wang X. (2014). Relation between emotion regulation and mental health: a meta-analysis review. *Psychol. Rep.* 114 341–362. 10.2466/03.20.PR0.114k22w4 24897894

[B46] Iacobucci D. (2010). Structural equations modeling: fit indices, sample size, and advanced topics. *J. Consum. Psychol.* 20 90–98. 10.1016/j.jcps.2009.09.003

[B47] Jamal M. Willem Van der Does A. J. Cuijpers P. Penninx B. W. (2012). Association of smoking and nicotine dependence with severity and course of symptoms in patients with depressive or anxiety disorder. *Drug Alcohol Depend.* 126 138–146. 10.1016/j.drugalcdep.2012.05.001 22633368

[B48] Jaremin B. Kotulak E. Starnawska M. (1997a). Comparative study of the death during sea voyages among Polish seamen and deep-sea and boat fishermen. *Bull. Inst. Marit. Trop. Med. Gdynia* 48 5–22. 9591146

[B49] Jaremin B. Kotulak E. Starnawska M. Mroziñski W. (1997b). Wojciechowski E. Death at sea: certain factors responsible for occupational hazard in Polish seamen and deep-sea fishermen. *Int. J. Occup. Med. Environ. Health* 10 405–416. 9575666

[B50] John U. Riedel J. Rumpf H. J. Hapke U. Meyer C. (2006). Associations of perceived work strain with nicotine dependence in a community sample. *Occup. Environ. Med.* 63 207–211. 10.1136/oem.2005.020966 16497864 PMC2078151

[B51] Joormann J. Gotlib I. H. (2010). Emotion regulation in depression: relation to cognitive inhibition. *Cogn. Emot.* 24 281–298. 10.1080/02699930903407948 20300538 PMC2839199

[B52] Juang L. P. Moffitt U. Kim S. Y. Lee R. M. Soto J. A. Hurley E. (2016). Cognitive reappraisal and expressive suppression: Links to racial-ethnic discrimination and adjustment among Latino/a and Asian-heritage college students. *J. Adolesc.* 53 21–33. 10.1016/j.adolescence.2016.08.012 27598799 PMC7891868

[B53] Kaerlev L. Dahl S. Nielsen P. S. Olsen J. Hannerz H. Jensen A. (2007). Hospital contacts for chronic diseases among danish seafarers and fishermen: a population-based cohort study. *Scand. J. Public Health* 35 481–489. 10.1080/14034940701267385 17852993

[B54] Kashdan T. B. Barrios V. Forsyth J. P. Steger M. F. (2006). Experiential avoidance as a generalized psychological vulnerability: comparisons with coping and emotion regulation strategies. *Behav. Res. Ther.* 44 1301–1320. 10.1016/j.brat.2005.10.003 16321362

[B55] Khaled S. M. Bulloch A. Exner D. V. Patten S. B. (2009). Cigarette smoking, stages of change, and major depression in the Canadian population. *Can. J. Psychiatry* 54 204–208. 10.1177/070674370905400309 19321025

[B56] Lapeyre-Mestre M. Sulem P. Niezborala M. Ngoundo-Mbongue T. B. Briand-Vincens D. Jansou P. (2004). Taking drugs in the working environment: a study in a sample of 2106 workers in the Toulouse metropolitan area. *Therapie* 59 615–623. 10.2515/therapie:2004107 15789825

[B57] Larsen J. K. Vermulst A. A. Eisinga R. English T. Gross J. J. Hofman E. (2012). Social coping by masking? Parental support and peer victimization as mediators of the relationship between depressive symptoms and expressive suppression in adolescents. *J. Youth Adolesc.* 41 1628–1642. 10.1007/s10964-012-9782-7 22739935 PMC3492695

[B58] Lee J. S. Joo E. J. Choi K. S. (2013). Perceived stress and self-esteem mediate the effects of work-related stress on depression. *Stress Health* 29 75–81. 10.1002/smi.2428 22610597

[B59] Lerman C. Audrain J. Orleans C. T. Boyd R. Gold K. Main D. (1996). Investigation of mechanisms linking depressed mood to nicotine dependence. *Addict. Behav.* 21 9–19. 10.1016/0306-4603(95)00032-1 8729703

[B60] Lin T. C. Lin H. S. Cheng S. F. Wu L. M. Ou-Yang M. C. (2016). Work stress, occupational burnout and depression levels: a clinical study of paediatric intensive care unit nurses in Taiwan. *J. Clin. Nurs.* 25 1120–1130. 10.1111/jocn.13119 26914523

[B61] Loprinzi P. D. Walker J. F. Kane C. Cardinal B. J. (2014). Physical activity moderates the association between nicotine dependence and depression among U.S. smokers. *Am. J. Health Promot.* 29 37–42. 10.4278/ajhp.130301-QUAN-92 24200248

[B62] Lyons M. Hitsman B. Xian H. Panizzon M. S. Jerskey B. A. Santangelo S. (2008). A twin study of smoking, nicotine dependence, and major depression in men. *Nicotine Tob. Res.* 10 97–108. 10.1080/14622200701705332 18188750

[B63] Magnavita N. Fileni A. (2014). Association of work-related stress with depression and anxiety in radiologists. *Radiol. Med.* 119 359–366. 10.1007/s11547-013-0355-y 24297590

[B64] Makambi K. H. Williams C. D. Taylor T. R. Rosenberg L. Adams-Campbell L. L. (2009). An assessment of the CES-D scale factor structure in black women: the Black Women’s Health Study. *Psychiatry Res.* 168 163–170. 10.1016/j.psychres.2008.04.022 19501414 PMC2704501

[B65] Manley M. J. de Jonge P. Kershaw T. S. Desai R. A. Lin H. Kasl S. V. (2009). Association of major depression with subtypes of nicotine dependence found among adult daily smokers: a latent class analysis. *Drug Alcohol Depend.* 104 126–132. 10.1016/j.drugalcdep.2009.04.013 19505773 PMC3881368

[B66] Masumoto K. Taishi N. Shiozaki M. (2016). Age and gender differences in relationships among emotion regulation, mood, and mental health. *Gerontol. Geriatr. Med.* 2:2333721416637022. 10.1177/2333721416637022 28138490 PMC5119800

[B67] McKenzie M. Olsson C. A. Jorm A. F. Romaniuk H. Patton G. C. (2010). Association of adolescent symptoms of depression and anxiety with daily smoking and nicotine dependence in young adulthood: findings from a 10-year longitudinal study. *Addiction* 105 1652–1659. 10.1111/j.1360-0443.2010.03002.x 20707783

[B68] Melchior M. Caspi A. Milne B. J. Danese A. Poulton R. Moffitt T. E. (2007). Work stress precipitates depression and anxiety in young, working women and men. *Psychol. Med.* 37 1119–1129. 10.1017/S0033291707000414 17407618 PMC2062493

[B69] Meszaros V. Cserhati Z. Olah A. Perczel Forintos D. Adam S. (2013). [Coping with work-related stress in health care professionals—strategies for the prevention of burnout and depression]. *Orv. Hetil.* 154 449–454. 10.1556/OH.2013.29572 23506801

[B70] Moore S. A. Zoellner L. A. Mollenholt N. (2008). Are expressive suppression and cognitive reappraisal associated with stress-related symptoms? *Behav. Res. Ther.* 46 993–1000. 10.1016/j.brat.2008.05.001 18687419 PMC2629793

[B71] Muthén L. Muthén B. (1998–2010). *Mplus User’s Guide*, 7th Edn. Los Angeles, CA: Muthen & Muthen.

[B72] Ndiaye M. Hane A. A. Ndir M. Ba O. Diop-Dia D. Kandji M. (2001). Smoking habits among physicians in Dakar. *Rev. Pneumol. Clin.* 57 (1 Pt 1), 7–11. 11373598

[B73] Norberg M. M. Ham L. S. Olivier J. Zamboanga B. L. Melkonian A. Fugitt J. L. (2016). Pregaming and emotion regulation’s relationship to alcohol problems in college students: a cross-sectional study. *Subst. Use Misuse* 51 1024–1033. 10.3109/10826084.2016.1152498 27070827

[B74] Ochsner K. N. Bunge S. A. Gross J. J. Gabrieli J. D. E. (2002). Rethinking feelings: an fMRI study of the cognitive regulation of emotion. *J. Cogn. Neurosci.* 14 1215–1229. 10.1162/089892902760807212 12495527

[B75] Ong A. D. Walsh D. A. (2001). Nicotine dependence, depression, and the moderating role of goal cognitions. *Psychol. Addict. Behav.* 15 252–254. 10.1037/0893-164X.15.3.252 11563804

[B76] Pedersen W. von Soest T. (2009). Smoking, nicotine dependence and mental health among young adults: a 13-year population-based longitudinal study. *Addiction* 104 129–137. 10.1111/j.1360-0443.2008.02395.x 19133898

[B77] Percin F. Akyol O. Davas A. Saygi H. (2012). Occupational health of Turkish Aegean small-scale fishermen. *Occup. Med.* 62 148–151. 10.1093/occmed/kqr181 22113895

[B78] Pflanz S. E. Ogle A. D. (2006). Job stress, depression, work performance, and perceptions of supervisors in military personnel. *Mil. Med.* 171 861–865. 10.7205/MILMED.171.9.861 17036607

[B79] Phillips M. L. Ladouceur C. D. Drevets W. C. (2008). A neural model of voluntary and automatic emotion regulation: implications for understanding the pathophysiology and neurodevelopment of bipolar disorder. *Mol. Psychiatry* 13 833–857. 10.1038/mp.2008.65 18574483 PMC2745893

[B80] Priyanka R. Rao A. Rajesh G. Shenoy R. Pai B. M. (2016). Work-associated stress and nicotine dependence among law enforcement personnel in Mangalore, India. *Asian Pac. J. Cancer Prev.* 17 829–833. 10.7314/APJCP.2016.17.2.829 26925687

[B81] Radloff L. S. (1977). The CES-D scale: a self-report depression scale for research in the general population. *Appl. Psychol. Meas.* 1 385–401. 10.1177/014662167700100306 26918431

[B82] Richmond S. Hasking P. Meaney R. (2017). Psychological distress and non-suicidal self-injury: the mediating roles of rumination, cognitive reappraisal, and expressive suppression. *Arch. Suicide Res.* 21 62–72. 10.1080/13811118.2015.1008160 26645313

[B83] Russell M. A. H. Peto J. Patel U. A. (1974). The classification of smoking by factorial structure of motives. *J. R. Stat. Soc. A* 313–346. 10.2307/2344953

[B84] Sai L. Luo S. Ward A. Sang B. (2016). Development of the tendency to use emotion regulation strategies and their relation to depressive symptoms in Chinese adolescents. *Front. Psychol.* 7:1222. 10.3389/fpsyg.2016.01222 27597834 PMC4992678

[B85] Sandhu K. S. Arora V. Gupta N. Gupta P. Raja M. Mehta N. (2016). Association of occupational stress factors on nicotine dependence among patients visiting dental care unit of Indo-Tibetian border police force station in India. *Rocz. Panstw. Zakl. Hig.* 67 69–74. 26953584

[B86] Scherphof C. S. van den Eijnden R. J. Harakeh Z. Raaijmakers Q. A. Kleinjan M. Engels R. C. (2013). Effects of nicotine dependence and depressive symptoms on smoking cessation: a longitudinal study among adolescents. *Nicotine Tob. Res.* 15 1222–1229. 10.1093/ntr/nts260 23231824

[B87] Schmidt A. Neumann M. Wirtz M. Ernstmann N. Staratschek-Jox A. Stoelben E. (2010). The influence of occupational stress factors on the nicotine dependence: a cross sectional study. *Tob. Induc. Dis.* 8:6. 10.1186/1617-9625-8-6 20388193 PMC2865452

[B88] Son B. K. Markovitz J. H. Winders S. Smith D. (1997). Smoking, nicotine dependence, and depressive symptoms in the CARDIA Study. Effects of educational status. *Am. J. Epidemiol.* 145 110–116. 10.1093/oxfordjournals.aje.a009081 9006307

[B89] Song Y. Huang Y. Liu D. Kwan J. S. Zhang F. Sham P. C. (2008). Depression in college: depressive symptoms and personality factors in Beijing and Hong Kong college freshmen. *Compr. Psychiatry* 49 496–502. 10.1016/j.comppsych.2008.02.005 18702936

[B90] Soto J. A. Perez C. R. Kim Y. H. Lee E. A. Minnick M. R. (2011). Is expressive suppression always associated with poorer psychological functioning? A cross-cultural comparison between European Americans and Hong Kong Chinese. *Emotion* 11 1450–1455. 10.1037/a0023340 21707152

[B91] Sperberg E. D. Stabb S. D. (1998). Depression in women as related to anger and mutuality in relationships. *Psychol. Women Q.* 22 223–238. 10.1111/j.1471-6402.1998.tb00152.x

[B92] Szymanska K. Jaremin B. Rosik E. (2006). Suicides among Polish seamen and fishermen during work at sea. *Int. Marit. Health* 57 36–45. 17312692

[B93] Thomas T. K. Lincoln J. M. Husberg B. J. Conway G. A. (2001). Is it safe on deck? Fatal and non-fatal workplace injuries among Alaskan commercial fishermen. *Am. J. Ind. Med.* 40 693–702. 10.1002/ajim.10010 11757046

[B94] Trosclair A. Dube S. R. (2010). Smoking among adults reporting lifetime depression, anxiety, anxiety with depression, and major depressive episode, United States, 2005-2006. *Addict. Behav.* 35 438–443. 10.1016/j.addbeh.2009.12.011 20079577

[B95] Troy A. S. Wilhelm F. H. Shallcross A. J. Mauss I. B. (2010). Seeing the silver lining: cognitive reappraisal ability moderates the relationship between stress and depressive symptoms. *Emotion* 10 783–795. 10.1037/a0020262 21058843 PMC3278301

[B96] Wang L. Liu H. C. Li Z. Q. Du W. (2007). Reliability and validity of emotion regulation questionnaire Chinese revised version. *Chin. J. Health Psychol.* 15 503–505.

[B97] Wang X. D. Wang X. L. Ma H. (1999). “Russell reason for smoking questionnaire,” in *Rating Scales For Mental Health* ed. Hao W. (Beijing: Chinese Mental Health Journal Press) 360–363.

[B98] Wang Y. Chen X. Gong J. Yan Y. (2016). Relationships between stress, negative emotions, resilience, and smoking: testing a moderated mediation model. *Subst. Use Misuse* 51 427–438. 10.3109/10826084.2015.1110176 26894428 PMC4855524

[B99] Wieclaw J. Agerbo E. Mortensen P. B. Burr H. Tuchsen F. Bonde J. P. (2006). Work related violence and threats and the risk of depression and stress disorders. *J. Epidemiol. Commun. Health* 60 771–775. 10.1136/jech.2005.042986 16905721 PMC2566025

[B100] Wong S. Y. Chan D. Leung P. C. (2006). Depressive symptoms in middle-aged men: results from a household survey in Hong Kong. *J. Affect. Disord.* 92 215–220. 10.1016/j.jad.2006.01.027 16519946

[B101] Yang H. J. Soong W. T. Kuo P. H. Chang H. L. Chen W. J. (2004). Using the CES-D in a two-phase survey for depressive disorders among nonreferred adolescents in Taipei: a stratum-specific likelihood ratio analysis. *J. Affect. Disord.* 82 419–430. 10.1016/j.jad.2004.04.008 15555693

[B102] Yu H. Tao Y. J. Pan H. X. Jiang N. N. Ma H. Y. (2014). Development of mental stressor investigation questionnaire among the crew of naval ships. *J. Prev. Med. Chin. Peoples Liberation Army.* 32 119–121.

[B103] Yuan J. Liu Y. Ding N. Yang J. (2014). The regulation of induced depression during a frustrating situation: benefits of expressive suppression in Chinese individuals. *PLoS One* 9:e97420. 10.1371/journal.pone.0097420 24827934 PMC4020863

[B104] Zeigelboim B. S. da Silva T. P. Carvalho H. de Brito Malucelli D. A. de Oliveira Goncalves C. G. Albizu E. J. (2014). Otoneurologic findings in a fishermen population of the state of santa catarina: preliminary study. *Int. Arch. Otorhinolaryngol.* 18 6–10. 10.1055/s-0033-1358584 25992055 PMC4296937

[B105] Zeigelboim B. S. Santos da Carvalho H. A. Goncalves C. G. Albizu E. J. Marques J. M. (2015). Otoneurological symptoms in Brazilian fishermen exposed over a long period to carbon monoxide and noise. *Noise Health* 17 300–307. 10.4103/1463-1741.165053 26356372 PMC4900498

[B106] Zhao Y. Zhao G. (2015). Emotion regulation and depressive symptoms: examining the mediation effects of school connectedness in Chinese late adolescents. *J. Adolesc.* 40 14–23. 10.1016/j.adolescence.2014.12.009 25600512

